# Medicine Men of the World

**Published:** 1887-11-12

**Authors:** 


### MEDICINE MEN OF THE WORLD.

**By the Man in the Crowd.**

### III.—MEDICINE MEN IN SAVAGE LANDS.

(*Continued from p.* 82, *Vol. III.*)

Medical practice in savage lands must of course always be more or less of the nature of quackery, and almost equally of course a good many of the more intelligent of savages must be perfectly well aware of it. They cannot but be quite conscious of the humbug of much of the medicine man's procedure, and now and again some of the more unscrupulous have managed to improve upon the orthodox practice of their tribes and make a reputation all their own. There is, for instance, among the Omaha Indians a tradition of a terrible chief whose repute as a medicine man gave him the most despotic power over his people. Anyone incurring his displeasure in the slightest degree would have his death predicted within a given time, and as the prediction invariably came true, this fearsome chieftain came to be regarded with an awe comparable only with that inspired by a supernatural being. The secret of his gift of prophecy was that he had met with a white man who had supplied him with arsenic. He could not cure, but he could kill, and by the crafty exercise of this power, easily made a short cut to eminence as a medicine man.

But, generally speaking, entrance to the medical profession all over the world is not easy, and those who adopt it are held in great estimation, partly from a genuine belief in their power to afford relief in times of sickness and suffering, and partly from the dealings with the supernatural world with which they are very commonly accredited. Among some uncivilised tribes every man seems to be his own doctor. Thus, the inhabitants of Samoa, whenever they find themselves indisposed, do not seek, or at any rate till their intercourse with Europeans perhaps gave them one or two new ideas, they did not seek any medicine man. They simply took a dose of the most disgusting filth they could find, in order to make themselves sick. This idea is nasty enough even for a savage, but it is interesting to note that, savages though they were, the practice appears to have indicated some recognition of the condition of the stomach as a very general source of disorder. The Samoans were thus seemingly in possession of one of the great central truths of modern scientific medicine. Could it possibly be that the aborigines of Northern Australia had a dim perception of Harvey's great revelation? Their specific for all sorts of ailments was to tightly bandage any affected portion of their bodies in order to prevent the spread of the malady. That a great many uncivilised peoples have from time immemorial understood something of the benefits which modern medicine has discovered in the Turkish bath is very well known. The Indian sudatory is an object very familiar to travellers. It is usually situated near a village, and on the banks of a stream, and is generally constructed of skins tightly sewn together. An Indian who feels hardly up to the mark will sit in one of these structures quite naked, while his squaw will bring in from a furnace close at hand a series of red-hot stones for him to pour out water upon, and thus give himself a thorough steaming within and without—just such a sweltering, in fact, as we clever people pay our three-and-sixpence for in the calidarium of a London Turkish bath. Having thus induced a profuse perspiration—the steam, by the way, sometimes being made to pass through mats of aromatic herbs supposed to have medicinal virtue—the patient rushes out to the river and takes a "header," scrambling out the next minute to hasten home, wipe himself dry, wrap himself in his buffalo cloak, and take a refreshing nap with his feet to the fire.

A man, however, cannot always be his own doctor. There are occasions on which he must at least call in his friends. A Polynesian who has the misfortune to dislocate a limb will summon them to his assistance and not any professional medicine man whose hocus-pocus will hardly avail to get a bone into its socket, whatever it may do for driving out an evil spirit from the patient who has a bad attack of colic. Friends and neighbours gather around; one of them will secure his head by holding it between the knees, while the others will twist and squeeze and pommel the disjointed member in any way that seems to be required until it slips into its place again. Dentistry is sometimes amateur and sometimes professional. Mr. Galton tells us that on one occasion, while in the Damarus, he had an opportunity of witnessing a native professor extract a tooth. "Tying one end of a sheep's sinew round the offending grinder, the manipulator fastened to the other a stout stick, upon which he proceeded to wind up the cat-gut, until, by pressing with his whole force against the jaw, and gaining all the leverage which this expedient allowed, the tusk was torn up by the fangs—or something else (it mattered little) yielded to the barbarous strain. For the rest of the day," says the traveller, "the wretched sufferer was seen sitting with his head between his knees and his hands against his temples."

Not only have savage peoples here and there medical practices very near akin to those obtaining among ourselves, but it seems to be a fact, that even the beneficence of the anæsthetic has been by no means unknown to some of them. Thus our traveller affirms, that a medicine man of the Ittots undertook the cure of a bad case of rheumatism, and the first thing he did was to administer a powerful narcotic, which soon rendered the patient quite insensible. Alas! poor fellow, he had better have kept awake. Having got the victim entirely under his thumb, the surgeon began with a sharp crease to slash and cut his skin from head to foot, until he presented the appearance of having been flayed alive, and was rendered incapable of either eating, drinking, or even speaking without the greatest difficulty.

The trephining process, which quite recently has made some little noise in the surgical world, is, it appears, an old thing in some less scientific lands. The native medicine men in the Island of Borabora are said to have among them a custom of clearing away any fragments of broken bone found in the cranium of a brave who has fallen under a shillelah in a fight, and replacing the scalp by a neat patch of cocoa-nut shell, stretching over it the scalp skin as far as practicable. In cases where the brain as well as the skull was injured, these daring operators were wont to remove the damaged portions and substitute an equivalent weight of cerebral matter from a pig's head. Strange to say, in spite of all precautions, the patient usually died in a condition of inflammation and frenzy!

(*To be continued.*)

